# Immunophenotype Discovery, Hierarchical Organization, and Template-Based Classification of Flow Cytometry Samples

**DOI:** 10.3389/fonc.2016.00188

**Published:** 2016-08-31

**Authors:** Ariful Azad, Bartek Rajwa, Alex Pothen

**Affiliations:** ^1^Lawrence Berkeley National Laboratory, Computational Research Division, Berkeley, CA, USA; ^2^Bindley Bioscience Center, Purdue University, West Lafayette, IN, USA; ^3^Department of Computer Science, Purdue University, West Lafayette, IN, USA

**Keywords:** flow cytometry, clusters, meta-clusters, template, matching, classification

## Abstract

We describe algorithms for discovering immunophenotypes from large collections of flow cytometry samples and using them to organize the samples into a hierarchy based on phenotypic similarity. The hierarchical organization is helpful for effective and robust cytometry data mining, including the creation of collections of cell populations’ characteristic of different classes of samples, robust classification, and anomaly detection. We summarize a set of samples belonging to a biological class or category with a statistically derived template for the class. Whereas individual samples are represented in terms of their cell populations (clusters), a template consists of generic meta-populations (a group of homogeneous cell populations obtained from the samples in a class) that describe key phenotypes shared among all those samples. We organize an FC data collection in a hierarchical data structure that supports the identification of immunophenotypes relevant to clinical diagnosis. A robust template-based classification scheme is also developed, but our primary focus is in the discovery of phenotypic signatures and inter-sample relationships in an FC data collection. This collective analysis approach is more efficient and robust since templates describe phenotypic signatures common to cell populations in several samples while ignoring noise and small sample-specific variations. We have applied the template-based scheme to analyze several datasets, including one representing a healthy immune system and one of acute myeloid leukemia (AML) samples. The last task is challenging due to the phenotypic heterogeneity of the several subtypes of AML. However, we identified thirteen immunophenotypes corresponding to subtypes of AML and were able to distinguish acute promyelocytic leukemia (APL) samples with the markers provided. Clinically, this is helpful since APL has a different treatment regimen from other subtypes of AML. Core algorithms used in our data analysis are available in the flowMatch package at www.bioconductor.org. It has been downloaded nearly 6,000 times since 2014.

## 1 Introduction

Feature selection is the problem of identifying a representative set of features from a large dataset to construct a classification model. In this paper, we address a feature selection problem in flow cytometry (FC) data, with a view toward identifying features that would be used by clinicians in characterizing the presence of a disease or response to a drug or vaccine or some other stimulus. In FC data, such features correspond to cell populations in the immune system that express certain subsets of proteins while not expressing others. Hence, we propose a method to identify these immunophenotypes in FC data and to use them for organizing a collection of samples into a hierarchy for classification purposes.

Flow cytometry (FC) is a mature technology for measuring the morphology (from scattering) and the expression of multiple biomarkers (from fluorescence) at the single-cell level (1). Each FC sample consists of hundreds of thousands or more of such single-cell measurements, and a study could consist of hundreds of samples from different individuals at different time points under different experimental conditions (2). Analyzing large, high-dimensional, noisy, and heterogeneous data collections generated by modern flow cytometers has become a challenge for human operators. Researchers have responded to this challenge by developing a number of automated tools that have become popular to analyze FC data (2–5).

Unlike most prior work that analyzes one sample at a time, here we process heterogeneous FC samples collectively by summarizing a group of similar samples with representative templates. We organize an FC data collection in a hierarchical data structure that supports the identification of immunophenotypes relevant to clinical diagnosis. A robust template-based classification scheme is also developed, but the primary focus of the paper lies in the discovery of phenotypic signatures and inter-sample relationships in FC data collection.

Like other branches of biotechnology, FC has experienced an unprecedented expansion in the last decade. Current fluorescence-based technology supports the measurements of up to twenty proteins simultaneously in each cell (6), whereas atomic mass cytometry systems such as CyTOF (7) can measure more than forty markers per cell. When thousands of such high-dimensional samples are produced in an experiment, researchers have no other alternative but to automate the data analysis. Considering the complexity of FC data and the diversity of experiments, the analysis process is often divided into smaller steps for convenience in solving subproblems independently. Even though there is no consensus among scientists on a standard set of analysis steps, existing literature repeatedly used the following steps: (1) spectral unmixing or compensation to correct the effect of overlapping fluorescence channels and autofluorescence (8–10), (2) data transformation and normalization (11–16), (3) gating or clustering to identify cell populations (2, 3, 17, 18), (4) registering cell clusters across samples to establish correspondence (3, 15, 19), and (5) categorizing samples into distinct classes and identifying phenotypes (2, 3, 20). A number of open-source R packages have been developed to solve different steps, such as flowCore, flowViz, flowClust, flowTrans, flowStats, and flowType packages in Bioconductor (21). Several other web-based platforms are also available for automated FC data analysis, such as ImmPort (22), GenePattern (4), and Cytobank (5).

The aforementioned analysis steps and their corresponding tools are often designed to process one sample at a time. This approach is adequate when the number of samples in an experiment is small or when samples are too heterogeneous to be analyzed collectively. By contrast, when a large number of samples belong to a few representative classes, another level of abstraction – in terms of meta-populations and templates – may simplify the analysis. Classifying samples based on a few representative templates has several advantages over techniques that directly compare pairs of samples, such as nearest-neighbor classifiers. It is more efficient since one compares a sample with a few templates only, rather than with all other samples; it is more robust since a template describes the features common to cell populations in several samples, while ignoring noise and small sample-specific variations. Previous work (3, 15, 19, 23) acknowledged the advantage of this collective approach and developed software to automate this process. In recent work, Lee et al. (23) proposed a joint clustering and matching (JCM) algorithm for simultaneous segmentation and alignment of cell populations across multiple samples. By modeling the inter-sample variation within a class with random-effects terms, they construct a parametric template for each class of samples. These templates are used to classify new samples with high accuracy (23), demonstrating the effectiveness of template-based classifiers in flow cytometry.

In this paper, we extend our prior work (24, 25) and that of other researchers by clearly defining steps in template-based data analysis and developing a generic framework for robust classification and immunophenotyping. After some initial preprocessing, we summarize a set of samples belonging to a biological class or category with a statistically derived template for the class. Whereas individual samples are represented in terms of their cell populations (clusters), a template consists of generic meta-populations (groups of homogeneous cell populations obtained from the samples in a class) that describe key phenotypes shared among all those samples. We differ from prior work by organizing the samples into a template tree that facilitates fast classification, creating templates at multiple levels in the hierarchy and updating templates dynamically. We provide efficient algorithms for data transformation and cluster validation, which precede the template-based analysis. Major components of the discussed tools are publicly available in two Bioconductor packages flowMatch and flowVS.

We demonstrate the utility of the template-based approach with two datasets: (1) a seven-dimensional healthy dataset consisting of 65 samples from five healthy individuals and (2) an acute myeloid leukemia (AML) dataset consisting 2,872 samples from 43 AML-positive patients and 316 healthy donors. In the first analysis, we show that hierarchical organization of samples efficiently captures different sources of within- and between-subject variations present in healthy immune systems. The second analysis employs templates and meta-clusters to discover immunophenotypes of AML and design a highly accurate classification scheme for AML. For this purpose, we have developed a scoring function that accounts for the diversity of the myeloid cell populations in the various subtypes of AML. In our analysis, we identified thirteen immunophenotypes corresponding to subtypes of AML and separated acute promyelocytic leukemia (APL) samples (APL has a different treatment regimen from other subtypes of AML). Earlier work on the same AML dataset has classified AML samples using methods such as nearest-neighbor classification, logistic regression, matrix relevance learning vector quantization, etc., but they have not identified these immunophenotypes; e.g., Ref. (26, 27).

We organize the rest of the paper as follows. Section 2 describes steps and the associated algorithms in the analysis of FC datasets. The analyses of healthy and AML datasets are presented in Sections 3 and 4, respectively. We conclude the paper in Section 4.

## 2 Steps in Analyzing FC Data

**Figure 1:**
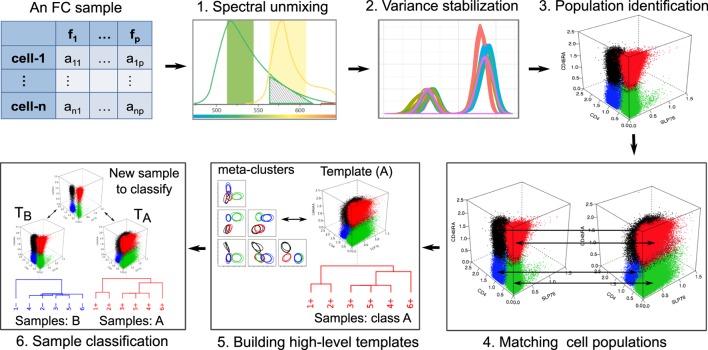
**In our view, six major steps are involved in the FC data analysis**. An FC sample is represented by an *n* × *p* matrix, where *n* is the number of cells and *p* is the number of features measured in each cell. (1) The overlap of two spectra (green and yellow) emitted by two fluorochromes, which must be unmixed to correctly reconstruct the signals. (2) The density plots of a marker from several samples of a dataset after transforming data to stabilize the variance. (3) Four cell populations (marked with different colors) identified by a clustering algorithm. (4) Matching population to register corresponding cell clusters across a pair of samples. (5) The hierarchical construction of a template from six samples belonging to the same class. (6) Classifying a sample based on its similarity with two templates.

Aside from some experiment-specific preprocessing, we logically divide FC data analysis into six distinct steps as shown in Figure 1. This division and ordering of work in FC data analysis is simply our view to tackle subproblems independently and develop algorithms to automated data analysis. Other researchers have divided these steps into smaller substeps (28), merged multiples steps into one (23), or ordered these steps differently based on the need of a particular experiment. In the rest of this section, we briefly discuss these steps.

### 2.1 Removing Unintended Cells

**Figure 2:**
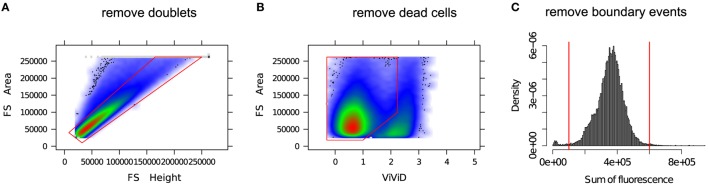
**Removing unintended events from an FC sample**. **(A)** Single intact cells (inside the red polygon gate) are separated from the doublets (outside of the red polygon gate). **(B)** A viability marker (ViViD) is used to remove dead cells (outside of the red polygon gate). **(C)** Cells emitting very low or very high fluorescence signals (outside of the red vertical lines) are removed as potential outlying events.

In the preprocessing phase, various unintended events such as doublets, dead cells, debris, etc. are removed from the FC data. A “doublet” is a pair of attached cells, which has a larger area but smaller height in the forward scatter (FS) channel relative to a single intact cell. Cell viability dyes, e.g., the amine reactive viability dyes ViViD and Aqua Blue, are often used to separate dead cells from live cells (29). Figure 2 shows several preprocessing and quality control steps used in a typical FC data analysis. Several other preprocessing steps are occasionally employed as part of quality control; for example, see the discussion in Ref. (30).

### 2.2 Spectral Unmixing (Compensation)

Flow cytometry measures the abundance of protein markers in a cell with the fluorescence intensities of fluorophore-conjugated antibodies bound to the target proteins. Because of the overlap of fluorescence spectra emitted by different fluorophores, a detector intended for a particular marker also captures partial emissions from other fluorophores. The correct signal at each detector is therefore recovered by a process called *spectral unmixing* or *compensation* (8, 9). Consider an FC system measuring the emission of *p* fluorophores with *p* detectors. Let **s** be a vector of the original signal emitted from the *p* fluorochromes and **o** be a vector of the observed signals at *p* detectors. Also, let **e** be a vector of length *p* measuring the amount of noise. We can construct a *p* × *p* spillover matrix M whose off-diagonal element M[*i, j*] denotes the fractional contribution of the *j*-th fluorochrome to the detector of the *i*-th fluorochrome. The diagonal elements of *M* represent the fraction of signals found in their designated channels. Each column of the spillover matrix adds to one. Then, the general form of the compensation system is given by **o** = M**s** + **e**. The goal of spectral unmixing is to calculate the actual signal vector **s**. The simplest and widely used algorithm performing compensation solves the system of linear equations involving the spillover matrix *M* and reconstructs **s** (9, 10). However, the accuracy of the reconstructed signal depends on the nature of errors generated by the fluorescence emission process and photo-electric circuitry of the flow cytometer. The error model can be approximated by a mixture of Poisson and Gaussian noise, and a more accurate compensation scheme is discussed in Ref. (10, 31).

### 2.3 Data Transformation and Variance Stabilization

After initial preprocessing and compensation, FC data are often transformed with non-linear functions [e.g., logarithm, hyperlog, biexponential, inverse hyperbolic sine (asinh), etc.] to project cell populations with normally distributed clusters – a choice that usually simplifies subsequent visual analysis (11–14). Recently, Finak et al. (15) used the maximum likelihood approach to normalize the cell populations, and Ray and Pyne (16) transformed each channel with the asinh function whose parameters were selected by the Jarque–Bera test of normality (a goodness-of-fit test of whether sample data have the skewness and kurtosis matching a normal distribution). While these approaches allow visual identification of cell populations within each sample, they are often inadequate when comparing cell populations across samples. This inadequacy arises from inhomogeneous variances in cell populations, a problem caused by the dependence of fluorescence variance on the mean fluorescence intensity. Due to such signal-variance dependence, a cell population with higher level of marker expressions (i.e., higher fluorescence emission) has higher variance than another population with relatively low level of marker expressions (i.e., low fluorescence emission). This inhomogeneity of within-population variance creates problems in extracting features uniformly and comparing cell populations with different levels of marker expressions. To address this problem, we select the parameters of traditional non-linear functions so that within-population variance is approximately stabilized, a process known as variance stabilization (VS) (32, 33).

We address the need for explicit VS in FC with a maximum likelihood (ML)-based method, called flowVS, which is built on top of a commonly used inverse hyperbolic since (asinh) transformation. The choice of asinh function is motivated by its success as a variance stabilizer for microarray data (33). flowVS stabilizes the within-population variances separately for each fluorescence channel *z* across a collection of *N* samples. After transforming *z* by asinh(*z*/*c*), where *c* is a normalization *cofactor*, flowVS identifies one-dimensional clusters (density peaks) in the transformed channel. Assume that a total of *m* 1-D clusters are identified from *N* samples with the *i*-th cluster having variance $\sigma_{i}^{2}$. Then, the asinh(*z*/*c*) transformation works as a variance stabilizer if the variances of the 1-D clusters are approximately equal, i.e., $\sigma_{1}^{2}∼\sigma_{2}^{2}∼\ldots ∼\sigma_{m}^{2}$. To evaluate the homogeneity of variance (also known as homoscedasticity), we use Bartlett’s likelihood-ratio test (34). From a wide range of cofactors, our algorithm selects one that minimizes Bartlett’s test statistics, resulting in a transformation with the best possible VS. In contrast to other transformation approaches, our algorithm applies the same transformation to corresponding channels in every sample. flowVS is therefore an explicit VS method that stabilizes within-population variances in each channel by evaluating the homoscedasticity of clusters with a likelihood-ratio test. flowVS is available as a free package in Bioconductor and is discussed in detail in a separate publication (35).

### 2.4 Cell Population Identification

A cell *population* or cell cluster is a homogeneous subset of cells in a sample with similar physical and fluorescence characteristics and thus biologically similar to other cells within the subset but distinct from those outside the subset. Traditionally, cell populations are identified by a manual process known as “gating,” where cell clusters are recognized in a collection of two-dimensional scatter plots (see Figure 7B for an example). However, with the ability to monitor a large number of protein markers simultaneously and to process a large number of samples with a robotic arm, manual gating is not feasible for high-dimensional or high-throughput FC data. To address the gating problem, researchers have proposed several automated clustering algorithms, such as FLAME (3), FLOCK (36), flowClust (17), flowMeans (18), SamSPECTRAL (37), SWIFT (38), etc. Aghaeepour et al. (2) provides a state-of-the-art summary of the field.

**Figure 3:**
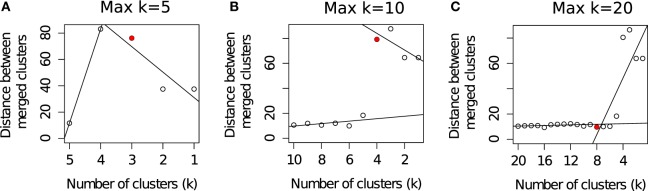
**Selecting the optimum number of cell populations in a sample from the HD dataset by the flowMeans package (18)**. The maximum number of clusters is set to: **(A)** 5 clusters (automatically selected by algorithm), **(B)** 10 clusters, and **(C)** 20 clusters. The optimum number of clusters is selected by detecting change point in the segmented regression lines and is shown with a red filled circle in each subfigure.

Different algorithms perform better for different FC datasets as was observed in a set of challenges organized by the FlowCAP consortium^1^ (2). Given the large number of algorithmic options, it is often difficult to select the best algorithm for a particular dataset. Even selecting the number of clusters for an algorithm is a non-trivial problem. For example, the flowMeans algorithm (18) starts with a relatively large number of clusters (Max *k*) and merges two closest clusters in each iteration. The algorithm then plots the distances between the merged clusters at each iteration and selects the optimum number of clusters where a sharp change in the segmented regression lines is observed. Figure 3 shows that the optimal number of clusters obtained by flowMeans depends heavily on the initial number of clusters. Therefore, we evaluate the quality of a clustering solution with several cluster validation methods (39, 40) and select the consensus number of clusters obtained from the validation criteria.

The cluster validation methods evaluate how well a given partition captures the natural structure of the data based on information intrinsic to the data alone. They can be used in selecting the optimum parameters for a clustering algorithm (e.g., the optimum number of clusters), as well as choosing the best algorithm for a dataset. To this end, we select an algorithm and the number of clusters *k* to be used with the algorithm by simultaneously optimizing five cluster validation methods: (1) average silhouette width, (2) Calinski–Harabasz (41), (3) Dunn’s index, (4) S_Dbw (42), and (5) Davies–Bouldin. The first three indices are maximized, and the last two indices are minimized (43). If different cluster validation methods disagree on the best clustering algorithm, we can use a consensus of several clustering solutions using an algorithm discussed in Section 2.6. We employ the selected algorithm (or the consensus of a collection of algorithms) with the chosen parameters to identify cell populations in an FC sample.

### 2.5 Registering Cell Populations across Samples

Population registration is a process of mapping phenotypically or functionally similar cell clusters across FC samples. When performed manually, cell populations are registered by visually mapping 2-D projections of clusters. However, manual registration is challenged by high dimensionality of FC data and large number of samples in a cohort because the number of manual comparisons grows quadratically with the number of clusters and samples. To expedite the registration process and to increase its accuracy, automated algorithms have been proposed recently (3, 15, 44). These algorithms can be categorized into two broad classes. In the first approach, the centers of different clusters are “meta-clustered” (cluster of clusters), and the clusters whose centers fall into the same meta-cluster are marked with the same label (15). The second approach computes a biologically meaningful distance or dissimilarity between each pair of clusters across samples, making use of the means and covariances of the clusters, and then matches similar clusters by using a combinatorial matching algorithm (3, 44). Here, we discuss an algorithm of the second type, called the mixed edge cover (MEC) algorithm (19, 44). The MEC algorithm uses a robust graph-theoretic framework to match a cluster from a sample to zero, one, or more clusters in another sample and thus solves the problem of missing or splitting clusters as well.

The MEC algorithm matches clusters with high similarity (low dissimilarity) while optimizing a global objective function. For this purpose, we calculate the dissimilarity between a pair of cell populations by the Mahalanobis distance between their distributions. Let *c*_1_(μ_1_, Σ_1_) and *c*_2_(μ_2_, Σ_2_) be two normally distributed clusters consisting of *n*_1_ and *n*_2_ cells, respectively. The Mahalanobis distance *d*(*c*_1_, *c*_2_) between the clusters is computed as follows:

(1) $$\begin{array}{c} d(c_{1},c_{2})=\frac{1}{2}{\left(\mu_{1}-\mu_{2}\right)}^{⊤}\Sigma_{p}^{-1}(\mu_{1}-\mu_{2}),\;\text{where}\\ \Sigma_{p}=((n_{1}-1)\Sigma_{1}+(n_{2}-1)\Sigma_{2})/(n_{1}+n_{2}-2). \end{array}$$

Other dissimilarity measures such as Kullback-Leibler (KL) divergence (45, 46), earth mover’s distance (EMD) (47, 48), chi-squared statistics, and Kolmogorov-Smirnov (KS) statistic can also be used instead of Mahalanobis distance.

#### 2.5.1 Overview of the Mixed Edge Cover Algorithm

Consider two FC samples *A* and *B* consisting of *k_a_* and *k_b_* cell populations such that $A=\{a_{1},a_{2},\ldots ,a_{k_{a}}\}$, and $B=\{b_{1},b_{2},\ldots ,b_{k_{b}}\}$, where *a_i_* is the *i*-th cluster from sample *A*, and *b_j_* is the *j*-th cluster from *B*. The mixed edge cover computes a mapping mec, of clusters across *A* and *B* such that mec(*a_i_*) ∈ *P*(*B*) and mec(*b_j_*) ∈ *P*(*A*), where *P*(*A*) (*P*(*B*)) is the power set of *A*(*B*). When a cluster *a_i_* (or *b_j_*) remains unmatched under mec, i.e., mec(*a_i_*) = ∅, we set *d*(*a_i_*, –) = λ, where the fixed cost λ is a penalty for leaving a vertex unmatched. We set λ to $\sqrt{p}$ so that a pair of clusters gets matched only if the average squared deviation across all dimensions is less than one. The cost of a mixed edge cover mec is the sum of the dissimilarities of all pairs of matched clusters and the penalties due to the unmatched clusters. A minimum cost mixed edge cover is a mixed edge cover with the minimum cost. We use this minimum cost as the dissimilarity *D*(*A, B*) between a pair of samples *A* and *B*:

(2) $$\underset{\begin{array}{c} \text{mixed}\;\text{edge}\\ \text{covers},\;\mathrm{mec} \end{array}}{\min}(\sum_{\begin{array}{c} 1\leq i\leq k_{a}\\ b_{j}\in \mathrm{mec}(a_{i}) \end{array}}d(a_{i},b_{j})\;+\sum_{\begin{array}{c} 1\leq i\leq k_{b}\\ a_{j}\in \mathrm{mec}(b_{i}) \end{array}}d(b_{i},a_{j})),$$

where *d*(*a_i_, b_j_*) is computed from equation (1). A minimum cost mixed edge cover can be computed by a modified minimum weight perfect matching algorithm in *O*(*k*^3^ log*k*) time, where *k* is the maximum number of clusters in a sample (44). The number of cell clusters *k* is typically small (less than 50 in typical experiments), and populations from a pair of samples can be registered in less than a second on a desktop computer. An implementation of the MEC algorithm is available in flowMatch package in Bioconductor.

The optimum cost of the MEC solution can be used as the dissimilarity measure between a pair of samples. This is similar in spirit with the R-metric, transfer distance or partition distance that computes the minimum number of augmentations and removals of cells needed to transform one partition into another (49). However, the partition distance can compare only two partitions (clusterings) of the same sample whereas our measure can work with partitions from the same sample, or from two different samples. In contrast to the partition distance metric that matches a cluster to at most one cluster, MEC is able to match a cluster to zero, one, or more clusters. Therefore, the MEC-based dissimilarity measure is more robust when the number of cell populations changes due to different conditions.

### 2.6 Creating Templates from a Collection of Samples

**Figure 4:**
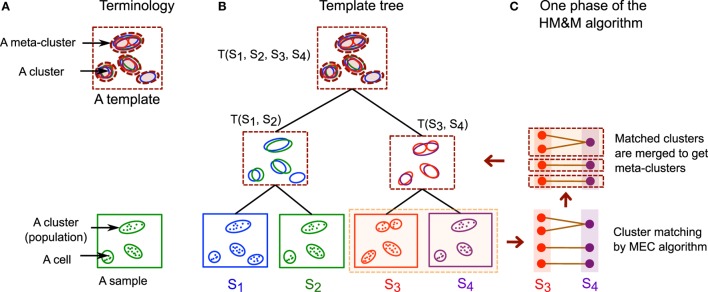
**(A)** Some of the terminologies used in this paper. A cell cluster or cell population is a group of cells expressing similar features, and an FC sample is a collection of cell clusters. A meta-cluster is a set of similar cell clusters from different samples, and a template is a collection of meta-clusters. Cells are denoted by dots, clusters by solid ellipses, and meta-clusters by dashed ellipses. **(B)** An example of a hierarchical template tree created from four hypothetical samples *S*_1_, *S*_2_, *S*_3_, and *S*_4_. A leaf node of the template tree represents a sample and an internal (non-leaf) node represents a template created from its children in the tree. The children could be templates if they are interior nodes or samples if they are leaves. **(C)** One step of the HM&M algorithm creating a template *T*(*S*_3_, *S*_4_) from a pair of samples. The algorithm first matches clusters (or meta-clusters) across samples (or templates) by the MEC algorithm and then merges the matched clusters to construct new meta-clusters.

When a collection of samples belongs to few representative classes and studying the overall features of these classes is the primary objective, we can summarize each class of samples with a statistically derived template (3, 15, 19). Here, different classes could represent multiple experimental conditions, disease status, time points, etc. Whereas individual samples are represented in terms of their cell populations (clusters), a template consists of generic meta-populations (group of homogeneous cell populations obtained from the samples in a class) that describe key phenotypes shared among all those samples. We summarize these concepts in Table 1 and also in Figure 4A.

**Table 1:** **Summary of terminology used in this paper**.

Terms	Meaning
Cell population (cluster)	A group of cells expressing similar features, e.g., T cells, B cells
Sample	A single biological sample characterized as a collection of cell populations
Meta-cluster	A set of biologically similar cell clusters from different samples
Template	A collection of meta-clusters from samples of the same class

Here, we describe an algorithm, called the hierarchical matching-and-merging (HM&M) algorithm, which organizes samples in a hierarchy and builds templates at the roots of trees representing the hierarchical organization of samples. The HM&M algorithm arranges a set of similar samples into a binary *template tree* data structure. A node in the tree represents either a sample (leaf node) or a template (internal node). In both cases, a node is characterized by a finite mixture of multivariate normal distributions each component of which is a cluster or meta-cluster. Figure 4 shows an example of a template tree created from four hypothetical samples, *S*_1_, *S*_2_, *S*_3_, and *S*_4_. The algorithm repeatedly merges the least dissimilar (most similar) pair of samples not already included in a template. The dissimilarity between a pair of samples is computed by the cost of an optimal mixed edge cover solution discussed earlier.

#### 2.6.1 Overview of the Template Construction Algorithm

Let a node *v_i_* (representing either a sample or a template) in the template tree consist of *k_i_* clusters or meta-clusters $c_{1}^{i},c_{2}^{i},\ldots ,c_{k_{i}}^{i}$. A node *v_i_* is called an “orphan” if it does not have a parent in the template tree. Consider *N* flow cytometry samples *S*_1_, *S*_2_, …, *S_N_* belonging to a class. Then, the HM&M algorithm for creating a template tree from these samples can be described by the following three steps.

1. *Initialization*: create a node *v_i_* for each of the *N* samples *S_i_*. Initialize all these nodes to the set of orphan nodes. Repeat the matching and merging steps until a single orphan node remains.
2. *Matching*: compute the dissimilarity *D*(*v_i_, v_j_*) between every pair of nodes *v_i_* and *v_j_* in the current orphan set with the mixed edge cover algorithm.
3. *Merging*: find a pair of orphan nodes (*v_i_, v*_j_) with minimum dissimilarity *D*(*v_i_, v_j_*) and merge them to create a new node *v_i_*. Let mec be a function denoting the mapping of clusters from *v_i_* to *v_j_*. That is, if $c_{x}^{i}\in v_{i}$ is matched to $c_{y}^{j}\in v_{j}$, then $c_{y}^{j}\in \mathrm{mec}(c_{x}^{i})$, where 1 ≤ *x* ≤ *k_i_* and 1 ≤ *y* ≤ *k_j_*. Create a new meta-cluster $c_{z}^{l}$ from each set of matched clusters, $c_{z}^{l}=\{c_{x}^{i}\cup \mathrm{mec}(c_{x}^{i})\}$. Let *k_l_* be the number of the new meta-clusters created above. Then, the new node *v_l_* is created as a collection of these newly created meta-clusters, i.e., $v_{l}=\{c_{1}^{l},c_{2}^{l},\ldots ,c_{k_{l}}^{l}\}$. The distribution parameters, $\left(\mu_{z}^{l},\Sigma_{z}^{l}\right)$, of each of the newly formed meta-clusters $c_{z}^{l}$ are computed from the distributions of the clusters participating in the meta-cluster. The height of *v_l_* is set to *D*(*v_i_, v_j_*). The node *v_l_* becomes the parent of *v_i_* and *v_j_*, and the set of orphan nodes is updated by including *v_l_* and deleting *v_i_* and *v_j_* from it. If there are orphan nodes remaining, we return to the matching step, and otherwise, we terminate.

When the class labels of samples are not known *a priori*, the roots of well-separated branches of tree give different class templates. However, if samples belong to the same class – as is the case for the AML dataset studied in this paper, the root of the template tree gives the class template. The HM&M algorithm requires *O*(*N*^2^) dissimilarity computations and *O*(*N*) merge operations for creating a template from a collection of *N* samples. Let *k* be the maximum number of clusters or meta-clusters in any of the nodes of the template tree. Then a dissimilarity computation takes *O*(*k*^3^ log *k*) time whereas the merge operation takes *O*(*k*) time when distribution parameters of the meta-clusters are computed by maximum likelihood estimation. Hence, the time complexity of the algorithm is *O*(*N*^2^*k*^3^ log *k*), which is *O*(*N*^2^) for bounded *k*. The complexity of the algorithm can be reduced to *O*(*N* log *N*) by avoiding the computation of all pairwise dissimilarities between the samples, for larger numbers of samples *N*, but we did not need to do this here.

#### 2.6.2 Comparisons among Different Algorithms for Creating Templates

Several other meta-clustering algorithms have also been discussed in the FC literature (3, 4, 15). FLAME (3) pools cluster centers (mean or medoid) from all samples of a class and clusters these centers to construct global meta-clusters. flowTrans (15) starts with seed meta-clusters and assigns each cluster to its nearest meta-cluster. Our algorithm is significantly different from both FLAME and flowTrans in several ways. First, FLAME and flowTrans both build a single template from samples of the same class. Therefore, they need to know the class labels of each sample, which is often not known in practice. In contrast, the HM&M algorithm identifies templates as the roots of the well-separated branches of the template tree in an *unsupervised* manner. Our approach also allows multiple templates to represent substantially different sub-classes within a single class, and therefore it is more flexible in covering sample diversity. Second, instead of clustering population centers, we optimally match populations across samples and then merge the matched clusters into meta-clusters (see Figure 4C for an example). Like FLAME, but unlike flowTrans, our approach allows a cluster to form a self-contained meta-cluster when it is significantly different from all other clusters. And finally, the hierarchical organization of samples (Figure 4B) provides additional flexibility in creating multi-layer templates, classifying samples, and updating templates dynamically.

### 2.7 Sample Classification Based on Templates

Given the inter-sample variations due to innate biological variability among individuals or Poisson and Gaussian noise from the FC measurements, a few templates can concisely represent a large cohort of samples by emphasizing their major characteristics while hiding statistical noise and unnecessary details. Thereby, overall changes across multiple conditions can be determined rigorously by comparing the cleaner and fewer class templates rather than the large number of noisy samples themselves (3, 19). We also show that the use of templates leads to efficient classification algorithms.

When the dataset consists of samples belonging to *m* classes, we build *m* templates, *T*_1_, *T*_2_, …, *T_m_*, where the *i*-th template *T_i_* represents samples of the *i*-th class. When we obtain a new sample *S*, we compute the dissimilarity *D*(*S, T_i_*) between *S* and every template *T_i_*. The new sample is predicted to belong to the class whose template it is most similar (least dissimilar) to. If a sample’s dissimilarity with the closest template is above a threshold, then it is not similar to any of the class templates, and we need to create a new class for this sample. The hierarchical organization of samples becomes handy in this situation when all samples are not available in the beginning. Then, samples are inserted in the already existing hierarchical data structure and update templates dynamically when new samples are classified (20). This approach is likely to improve the accuracy of future classification due to additional information gained from newly classified samples. The template-based classification is very fast because we need to compare a new sample only with *m* templates instead of all other samples and therefore requires *O*(*m*) dissimilarity computations instead of the *O*(*N*) dissimilarities that nearest-neighbor classification requires.

### 2.8 Classification Score of a Sample in the AML Dataset

Acute myeloid leukemia is a heterogeneous disease as it perturbs different cell populations differently depending on the subtype of AML; the progression of the disease and the immune profile of the affected individual also affect the cell populations. Hence, we develop a sophisticated scoring function to classify AML and other similar disease in order to improve the classification accuracy. The primary reason for the special scoring function is that an AML sample contains both normal (AML-unrelated) and AML-specific cell populations. The number of AML-specific cells can be smaller than the number of normal cells. Hence, AML-specific cell clusters are given higher weights than AML-unrelated cells. The scoring scheme developed for AML can also be applied to other datasets where the disease affects only a subset of cells.

Consider a sample *X* consisting of *k* cell populations S = {c_1_, c_2_, …, *c_k_*}, with the *i*-th cluster *c_i_* containing |*c_i_*| cells. Let *T*^−^ and *T*^+^ be the templates created from AML-negative (healthy) and AML-positive training samples, respectively. We now describe how to compute a score *f*(*X*) in order to classify the sample *X* to either the healthy class or the AML class.

The intuition behind the score is as follows. An AML sample contains two kinds of cell populations: (1) AML-specific myeloblasts and myeloid cells, and (2) AML-unrelated cell populations, such as lymphocytes. The former cell populations correspond to the immunophenotypes of AML-specific meta-clusters in the AML template, and hence, when we compute a mixed edge cover between the AML template and an AML sample, these clusters get matched to each other. (Such clusters in the sample do not match to any meta-cluster in the healthy template.) Hence we assign a positive score to a cluster in sample when it satisfies this condition, signifying that it is indicative of AML. AML-unrelated cell populations in a sample could match to meta-clusters in the healthy template, and also to AML-unrelated meta-clusters in the AML template. When either of these conditions is satisfied, a cluster gets a negative score, signifying that it is not indicative of AML. Since AML affects only the myeloid cell line and its progenitors, it affects only a small number of AML-specific cell populations in an AML sample. Furthermore, different subtypes of AML affect different cell types in the myeloid cell line. Hence, there are many more clusters common to healthy samples than there are AML-specific clusters common to AML samples. (This is illustrated later in Figures 12C,D.) Thus, we make the range of positive scores relatively higher than the range of negative scores. This scoring system is designed to reduce the possibility of a false negative (an undetected AML-positive patient), since this is more serious in the diagnosis of AML. Additional data such as chromosomal translocations and images of bone marrow from microscopy could confirm an initial diagnosis of AML from flow cytometry.

In the light of the discussion above, we need to identify AML-specific meta-clusters initially. Given the templates *T*^+^ and *T*^−^, we create a complete bipartite graph with the meta-clusters in each template as vertices, and with each edge weighted by the Mahalanobis distance between its endpoints. When we compute a minimum cost mixed edge cover in this graph, we will match meta-clusters common to both templates, and such meta-clusters represent non-myeloid cell populations that are not AML-specific. On the other hand, meta-clusters in the AML template *T*^+^ that are not matched to a meta-cluster in the healthy template *T*^−^ correspond to AML-specific meta-clusters. We denote such meta-clusters in the AML template *T*^+^ by the set *M*^+^.

Now, we can proceed to compare a sample against the template for healthy samples and the template for AML. We compute a minimum cost mixed edge cover between a sample *X* and the healthy template *T*^−^, and let mec^−^(*c_i_*) denote the set of meta-clusters in *T*^−^ mapped to a cluster *c_i_* in the sample *X*. Similarly, compute a minimum cost mixed edge cover between *X* and the AML template *T*^+^, and let mec^+^(*c_i_*) denote the set of meta-clusters in *T*^+^ mapped to a cluster *c_i_*. These sets could be empty if *c_i_* is unmatched in the mixed edge cover. We compute the average Mahalanobis distance between *c_i_* and the meta-clusters matched to it in the template *T*^−^, and define this as the dissimilarity *d*(*c_i_*, mec^−^(*c_i_*)). From the formulation of the mixed edge cover in (44), we have *d*(*c_i_*, mec^−^(*c_i_*)) ≤ 2λ. Hence, we define the *similarity* between *c_i_* and mec^−^(*c_i_*) as *s*(*c_i_*, mec^−^(*c_i_*)) = 2λ − *d*(*c_i_*, mec^−^(*c_i_*)). By analogous reasoning, the similarity between *c_i_* and mec^+^(*c_i_*) is defined as *s*(*c_i_*, mec^+^(*c_i_*)) = 2λ − *d*(*c_i_*, mec^+^(*c_i_*)).

The score of a sample is the sum of the scores of its clusters. We define the score of a cluster *c_i_, f*(*c_i_*), as the sum of two functions *f*^+^(*c_i_*) and *f*^−^(*c_i_*) multiplied with suitable weights. A positive score indicates that the sample belongs to AML, and a negative score indicates that it is healthy.

The function *f*^+^(*c_i_*) contributes a positive score to the sum if *c_i_* is matched to an AML-specific meta-cluster in the mixed edge cover between the sample *X* and the AML template *T*^+^, and a non-positive score otherwise. For the latter case, there are two subcases: If *c_i_* is unmatched in the mixed edge cover, it corresponds to none of the meta-clusters in the template *T*^+^, and we assign it a zero score. If *c_i_* is matched only to non-AML-specific meta-clusters in the AML template *T*^+^, then we assign it a small negative score to indicate that it likely belongs to the healthy class (recall that *k* is the number of clusters in sample *X*). Hence,

$$\begin{array}{l} f^{+}(c_{i})=\{\begin{array}{cc} s\left(c_{i},{\mathrm{mec}}^{+}(c_{i})\right), & \text{if}\;{\mathrm{mec}}^{+}(c_{i})\cap M^{+}\neq \emptyset ,\\ \begin{array}{l} -\frac{1}{k}\left[s(c_{i},{\mathrm{mec}}^{+}(c_{i}))\right],\\ \;\\ 0, \end{array} & \begin{array}{l} \text{if}\;{\mathrm{mec}}^{+}(c_{i})\cap M^{+}=\emptyset ,\\ \text{and}\;{\mathrm{mec}}^{+}(c_{i})\neq \emptyset ,\\ \text{if}\;{\mathrm{mec}}^{+}(c_{i})=\emptyset . \end{array} \end{array}\\ \; \end{array}$$

The function *f*^−^(*c_i_*) contributes a negative score to a cluster *c_i_* in the sample *X* if it is matched with some meta-cluster in the healthy template *T*^−^, indicating that it likely belongs to the healthy class. If it is not matched to any meta-cluster in *T*^−^, then we assign it a positive score λ. This latter subcase accounts for AML-specific clusters in the sample, or a cluster that is in neither template. In this last case, we acknowledge the diversity of cell populations in AML samples. Hence, we have

$$f^{-}(c_{i})=\{\begin{array}{ll} -\frac{1}{k}\left[s(c_{i},{\mathrm{mec}}^{-}(c_{i}))\right], & \text{if}\;{\mathrm{mec}}^{-}(c_{i})\neq \emptyset ,\\ \lambda , & \text{if}\;{\mathrm{mec}}^{-}(c_{i})=\emptyset . \end{array}$$

Finally, we define

(3) $$f(X)=\sum_{c_{i}\in X}\frac{\left|c_{i}\right|}{\left|X\right|}\frac{1}{2}(f^{+}(c_{i})+f^{-}(c_{i})).$$

Here, |*X*| is the number of cells in the sample *X*. The score of a cluster *c_i_* is weighted by the fractional abundance of cells in it.

## 3 Profiling Healthy Immune Systems of Five Individuals

### 3.1 The Healthy Dataset

In our first application, we analyze a healthy donor (HD) dataset measuring partial immune profiles of several healthy individuals in the presence of different sources of variations. In this experiment, peripheral blood mononuclear cells (PBMCs) were collected from five healthy individuals (denoted by “A,” “B,” “C,” “D,” and “E”) on up to four different days. Each sample was divided into five parts and analyzed through a flow cytometer at Purdue’s Bindley Bioscience Center. Thus, we have five technical replicates for each sample from a subject, totaling 65 FC samples. Each sample was stained using labeled antibodies against CD45, CD3, CD4, CD8, and CD19 protein markers. A selected 20 samples of this dataset is publicly available in healthyFlowData package in Bioconductor. The HD dataset includes three sources of variations: (1) technical or instrumental variation among replicates of the same sample, (2) within-subject temporal (day-to-day) variation, and (3) between-subject natural or biological variation. Multiple sources of variations make the HD dataset an ideal use case to demonstrate the effectiveness of automated tools and software discussed earlier.

### 3.2 Preprocessing and Spectral Unmixing

**Figure 5:**
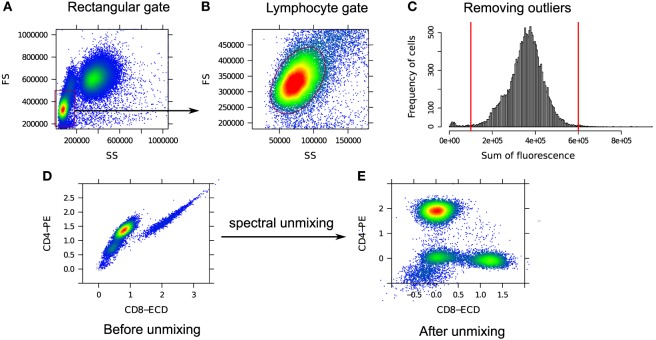
**(A)** A predefined rectangular gate (red rectangle on the lower left corner) denotes an approximate boundary for the lymphocytes. **(B)** Inside the rectangular gate, lymphocytes are identified as a dense and normally distributed region (red ellipse). **(C)** Outlying cells fall outside of a pair of predefined thresholds shown with the red vertical lines and are removed. **(D)** Correlated CD4 and CD8 expressions due to the spectral overlap between PE and ECD fluorochromes. **(E)** CD8 vs. CD4 expressions after spectral unmixing. The inverse hyperbolic sine (asinh) transformation is used in **(D,E)** for visualization.

We restrict our analysis to only lymphocyte cells that can be identified with a dense and normally distributed region within a predefined rectangular gate on the lower left corner of the forward scatter (FS) vs. side scatter (SS) scatter plot as shown in Figures 5A,B. To locate the normally distributed region within the rectangular gate, we used the norm2filter function from the flowCore package in R (50). From the lymphocyte population, we remove cells that are either too dim or too bright in terms of the total fluorescence emission. The former cells are potential debris since they do not express any marker and the latter cells are possibly doublets or noise from the flow cytometer. In Figure 5C, we eliminate the boundary events that fall outside of a pair of predefined thresholds shown with the red vertical lines. These thresholds are empirically selected from one sample and then applied to every other sample in the HD dataset.

Next, we compensate for the spectral overlap among fluorescence channels. Figure 5D shows the correlated expressions of CD4 and CD8 proteins in raw flow cytometry measurements. This correlation arises due to the overlap between the spectra of PE and ECD fluorochromes used to measure the expressions of CD4 and CD8 proteins, respectively. After unmixing the signals, the correlation is removed as can be seen in Figure 5E. Notice that different cell populations, e.g., CD4^+^ and CD8^+^ cells, are clearly identifiable after compensation.

### 3.3 Variance Stabilization

**Figure 6:**
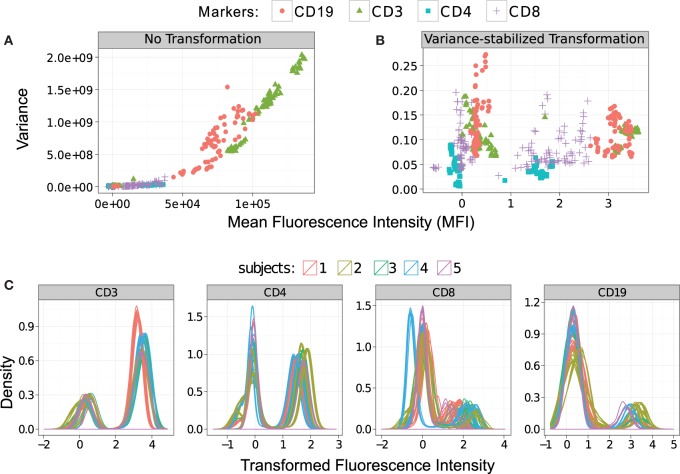
**Stabilizing the within-cluster variance for each channel of the HD dataset**. **(A)** Variances of the clusters increase monotonously with their means before the variances are stabilized. Clusters in each marker are shown with the same symbol and color. **(B)** Variances are approximately stabilized for each marker/channel after the data are transformed by the asinh function with the optimum cofactor. **(C)** Density of the variance-stabilized fluorescence channels are plotted where different subjects are denoted with different colors.

We stabilize the within-population variances in the HD dataset with the flowVS algorithm described in Section 2.3. For this purpose, we identify density peaks (also called 1-D clusters) in CD3, CD4, CD8, and CD19 markers/channels. For a particular channel, density peaks from all samples are pooled together, and variance of the corresponding 1-D clusters is stabilized by minimizing Bartlett’s statistics. Figure 6 plots the mean–variance relationship for every density peak before and after variance stabilization. In these subfigures, we show clusters from a channel with the same symbol and color. In Figure 6A, we observe a non-linear correlation between the variances and means of the clusters before variance stabilization. For example, CD3^+^ clusters (T lymphocytes) have much higher variance than CD3^−^ clusters (shown with green triangles in Figure 6A). After variance stabilization, the variances of the 1-D clusters become relatively stable as can be observed in Figure 6B. We plot the density of the variance-stabilized channels in Figure 6C, where different colors are used to denote samples from five different subjects. After variance stabilization, clusters with high and low marker expressions spread approximately equally in all samples, confirming the homogeneity of variances in one-dimensional clusters. For this dataset, the density curves from the same subjects are tightly grouped together, as expected. However, clusters from different subjects may not be well aligned due to the between-subject variations.

### 3.4 Cell Population Identification

**Figure 7:**
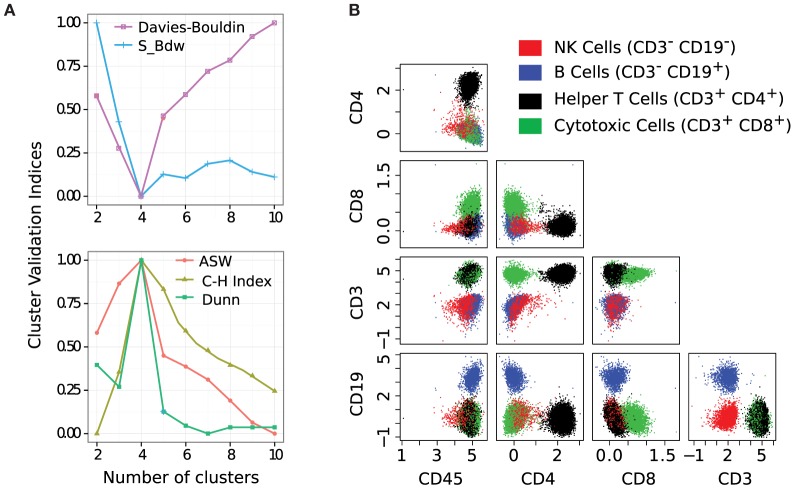
**(A)** Simultaneous optimization of five cluster validation criteria suggests that four cell populations are present in this sample. Here, three of the indices are maximized and two are minimized. **(B)** Bivariate projections of cell populations display four subsets of lymphocytes: red (natural killer cells), blue (B cells), black (helper T cells), and green (cytotoxic T cells). Each cell cluster is CD45^+^ since we pre-selected lymphocytes on the forward and side scatter channels.

We apply the k-means clustering algorithm to identify cell populations in each sample of the HD dataset. We have tested several other clustering algorithms, but k-means outperforms others according to several cluster validation methods. The optimum number of clusters is identified by the five cluster validation methods discussed in Section 2.4. In Figure 7A, we plot the values of the cluster validation indices (scaled to [0,1]) for a representative sample from the HD dataset. Three of the indices (Avg. Silhouette Width (ASW), Calinski-Harabasz (C-H), and Dunn) are maximized, and the rest (S_Dbw and Davies-Bouldin) are minimized. All validation methods unanimously indicate *k* = 4 as the optimum number of clusters for this sample. Therefore, we set the number of clusters *k* to four and assign cells to different clusters by applying the k-means algorithm.

The four clusters chosen by the k-means algorithm represent four subtypes of lymphocyte cells. These subtypes of cells are identified in the five-dimensional marker space. For visualization purposes, we show the cell populations by a collection of 2-D scatter plots in Figure 7B, where cell populations are shown in four different colors denoting (a) red: natural killer cells (CD45^+^CD3^−^CD19^−^), (b) blue: B cells (CD45^+^CD3^−^CD19^+^), (c) black: helper T cells (CD45^+^CD3^+^CD4^+^), and (d) green: cytotoxic T cells (CD45^+^CD3^+^CD8^+^). Here, every cluster is CD45^+^ because CD45 is a common leukocyte marker, and we pre-selected lymphocytes (a subtype of leukocytes) on the forward and side scatter channels.

### 3.5 Building Class Templates

**Figure 8:**
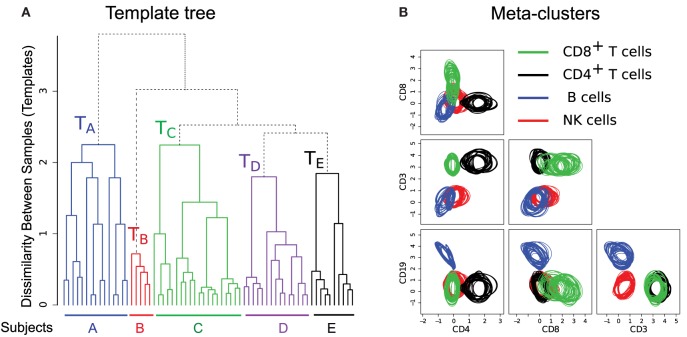
**(A)** The template tree created by HM&M algorithm from all samples of the HD dataset. Leaves of the dendrogram denote samples from five healthy individuals. An internal node represents a template, and the height of an internal node measures the dissimilarity between its left and right children. The sample-specific subtrees are drawn in different colors. **(B)** Bivariate projections of the combined template [the root of the tree in **(A)**] are drawn in terms of its meta-clusters. Here, each meta-cluster is represented by a homogeneous collection of cell clusters that are drawn with the 95th quantile contour lines. Clusters participating in a meta-cluster are drawn in same color.

To build templates, we organize the samples in a “template tree” shown in Figure 8A, where the leaf nodes denote samples from different healthy individuals. An internal node of the tree represents a template consisting of a homogeneous collection of meta-clusters. The height of an internal node measures the dissimilarity (the optimum cost of the mixed edge cover) between its left and right children. In Figure 8A, we draw branches joining samples from different subjects in five distinct colors. These branches create five disjoint subtrees whose roots represent templates of different healthy subjects (e.g., *T_A_* represents a template created from 15 samples from subject A). Here, the variations within a subject-specific template come from the effect of environment on individual immune systems on different days and technical variation in flow cytometry sample preparation and measurement. In contrast, the between-subject variations come from the natural biological variations in the healthy subjects. In this dataset, we observe more natural between-subject variations than the temporal and instrumental variations. Hence, samples from the five subjects create concise and well-separated templates representing immune profiles of different healthy individuals.

The hierarchical organization of samples has the ability of creating multi-layer templates. For the HD dataset, we observe three levels in the sample hierarchy in Figure 8A. In the lower level, a day-specific template is constructed from five replicates of a sample collected on a particular day from a subject. In the middle level, samples from a subject collected on different days create a subject-specific template denoted by the roots of colored subtrees in Figure 8A. Finally, in the top level, the root of the whole tree represents a combined template representing a healthy immune profile of these five subjects.

The combined template created at the root of the template tree in Figure 8A represents a healthy immune profile by preserving the common features of healthy individuals while removing subject-specific variations. This template consists of four meta-clusters denoting four sub-types of lymphocytes. Each meta-cluster is a homogeneous collection of cell populations from different samples. Figure 8B shows 2-D projections of these meta-clusters in terms of their participating clusters. The 95th quantiles of the clusters within a meta-cluster are shown in same color: (1) green – CD8^+^ T cells (CD45^+^CD3^+^CD8^+^), (2) black – CD4^+^ T cells (CD45^+^CD3^+^CD4^+^), (3) blue – B cells (CD45^+^CD3^−^CD19^+^), and (4) red – natural killer cells (CD45^+^CD3^−^CD19^−^). Figure 8B reveals that the distributions of clusters within a meta-cluster are relatively homogeneous. This healthy template can then be compared against disease templates for assessing the general effect of a disease on different cell types, which can lead to a robust and efficient disease diagnosis system.

### 3.6 Comparison with Alternative Approaches

When constructing a hierarchical organization of samples, we have many choices to make at different stages of the algorithm. For example, we can choose other dissimilarity measures between clusters than the Mahalanobis distance when we compute the mixed edge cover, and we can use another hierarchical clustering algorithm instead of the HM&M algorithm to organize samples. Here, we discuss how the other approaches perform on the HD dataset.

**Figure 9:**
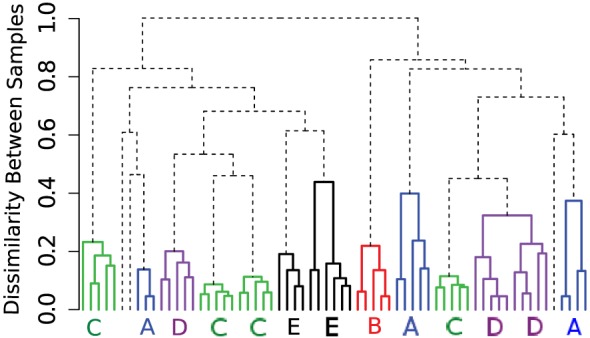
**Hierarchical organization of HD samples using the HM&M algorithm**. The Euclidean distance between cluster/meta-cluster centers is used when computing the mixed edge cover.

Beside the Mahalanobis distance, we have tested the Kullback–Leibler divergence and the Euclidean distance between cluster/meta-cluster centers as the dissimilarity between a pair of clusters or meta-clusters (these three options are available in the flowMatch package). We observe that the Kullback–Leibler divergence and Mahalanobis distance perform similarly for the HD dataset. However, when we use the Euclidean distance in the mixed edge cover computation, the template construction algorithm could not group samples from the same subjects, as shown in Figure 9. Euclidean distance does a reasonable job in grouping technical replicates (with two mistakes in subjects A and E). However, it fails to group samples collected on different days from the same subject because Euclidean distance does not consider the distribution of cells in a cluster or meta-cluster.

**Figure 10:**
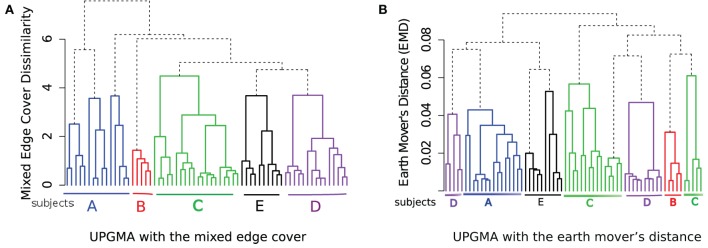
**Hierarchical organization of HD samples using the UPGMA algorithm using (A) the mixed edge cover (with Mahalanobis distance as the distance between clusters) and (B) the earth mover’s distance as the dissimilarity between every pair of samples**.

Next, we use the unweighted pair group method with arithmetic mean (UPGMA) hierarchical clustering algorithm to organize the samples in a hierarchy as shown in Figure 10. In Figure 10A, the dissimilarity between samples is computed by the mixed edge cover with Mahalanobis distance as the distance between clusters; and in Figure 10B, the dissimilarity between samples is computed by the earth mover’s distance (EMD) (47). To compute EMD, we used the flowFP package in Bioconductor to generate fingerprints of flow cytometry data, and then computed EMD by solving the minimum sum assignment problem using the clue package. UPGMA does a reasonable job in grouping samples from the same subject, albeit with few misplacements. For example, in Figure 10A, samples from subject A are split into two branches and in Figure 10B, samples from subject C and D are split into two branches. Therefore, the hierarchical matching and merging algorithm performs better than the UPGMA algorithm when discovering the relationship among FC samples. However, UPGMA or any other traditional hierarchical clustering algorithm cannot identify phenotypes shared among samples in different branches of the hierarchy because these algorithms only consider a dissimilarity measure between every pair of samples. By contrast, our template construction algorithm identifies phenotypes associated with meta-clusters and templates created from different classes of samples. These phenotypes provide key insight on the overall properties of the samples and supply valuable information for biological validation of the classification algorithms. In the next section, we discuss how this ability can be used to discover immunophenotypes of complex diseases such as AML.

## 4 Discovering Immunophenotypes of Acute Myeloid Leukemia

### 4.1 Background on AML

Acute myeloid leukemia is a disease of myeloid stem cells that differentiate to form several types of cells in the blood and marrow. It is characterized by the profusion of immature myeloid cells, which are usually prevented from maturing due to the disease. The myeloid stem cell differentiates in several steps to form myeloblasts and other cell types in a hierarchical process. This hierarchical differentiation process could be blocked at different cell types, leading to the multiple subtypes of AML. Eight different subtypes of AML based on cell lineage are included in the French–American–British Cooperative Group (FAB) classification scheme (51). [A different World Health Organization (WHO) classification scheme has also been published].

### 4.2 The AML Dataset

We use an AML dataset from the DREAM6/FlowCAP2 challenge of 2011 (publicly available at http://flowrepository.org/). The dataset consists of FC measurements of peripheral blood or bone marrow aspirate collected from 43 AML-positive patients and 316 healthy donors over a 1-year period. Each patient sample was subdivided into eight aliquots (“tubes”) and analyzed with different biomarker combinations, five markers per tube (most markers are proteins). In addition to the markers, the forward scatter (FS) and side scatter (SS) of each sample were also measured in each tube. Hence, we have 359 × 8 = 2,872 samples, and each sample is seven-dimensional (five markers and the two scatters). The disease status (AML/healthy) of 23 AML patients and 156 healthy donors are provided as training set, and the challenge is to determine the disease status of the rest of the samples, 20 AML and 157 healthy, based only on the information in the training set. Since samples are already compensated and logarithmically transformed, we omit these steps in our analysis. We also omit Tubes 1 and 8 because they are isotype and unstained controls, respectively.

### 4.3 Cell Populations in Healthy and AML Samples

**Figure 11:**
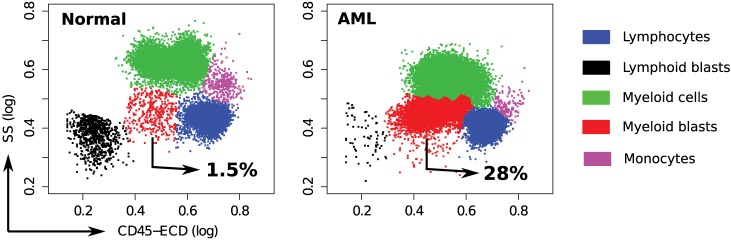
**Cell types identified on the side scatter (SS) and CD45 channels for a healthy and an AML-positive sample**. Cell populations are discovered in the seven-dimensional samples with the clustering algorithm and then projected on these channels for visualization. A pair of clusters denoting the same cell type is marked with the same color. The proportion of myeloid blast cells (shown in red) increases significantly in the AML sample.

In each tube, we identify cell populations in the samples using the k-means clustering algorithm. As in the analysis of the HD dataset, the number of clusters is selected by simultaneous optimization of five cluster validation methods discussed in Section 2.4. Each sample contains five major cell types that can be seen when cell clusters are projected on the side scatter (SS) and CD45 channels, as depicted in Figure 11. (Blast cells are immature progenitors of myeloid cells or lymphocytes.) The side scatter measures the granularity of cells, whereas CD45 is variably expressed by different white blood cells (leukocytes). AML is initially diagnosed by rapid growth of immature myeloid blast cells with medium SS and CD45 expressions (52) marked in red in Figure 11. According to the WHO guidelines, AML is initially confirmed when the sample contains more than 20% blasts. This is the case for all, except one of the AML samples in the DREAM6/FlowCAP2 training set, and the exception will be discussed later.

### 4.4 Healthy and AML Templates

**Figure 12:**
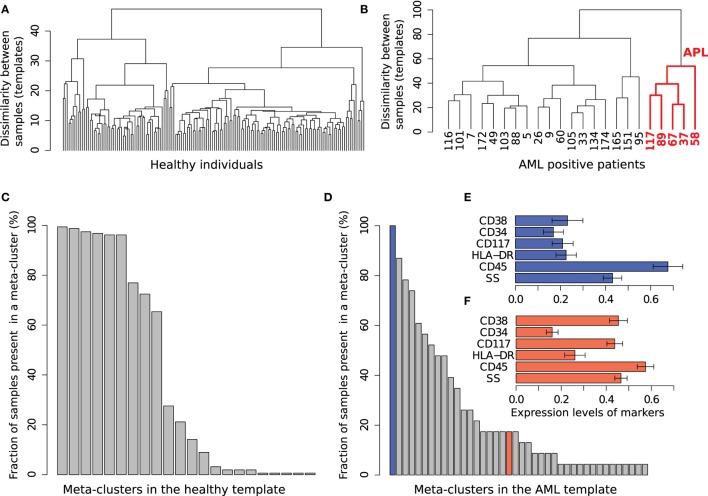
**The healthy and AML templates created from Tube 6**. **(A)** The template tree created from 156 healthy samples in the training set. **(B)** The template tree created from 23 AML samples in the training set. Samples in the red subtree exhibit the characteristics of acute promyelocytic leukemia (APL) as shown in **(F)**. **(C)** Fraction of 156 healthy samples present in each of the 22 meta-clusters in the healthy template. Nine meta-clusters, each of them shared by at least 60% of the healthy samples, form the core of the healthy template. **(D)** Fraction of 23 AML samples present in each of the 40 meta-clusters in the AML template. The AML samples, unlike the healthy ones, are heterogeneously distributed over the meta-clusters. **(E)** The expression levels of markers in the meta-cluster shown with blue bar in **(D)**. [Each horizontal bar in **(E,F)** represents the average expression of a marker and the error bar shows its SD]. This meta-cluster represents lymphocytes denoted by medium SS and high CD45 expression and therefore does not express the AML-related markers measured in Tube 6. **(F)** Expression of markers in a meta-cluster shown with red bar in **(D)**. This meta-cluster denotes myeloblast cells as defined by the SS and CD45 levels. This meta-cluster expresses HLA-DR^−^CD117^+^CD34^−^CD38^+^, a characteristic immunophenotype of APL. Five AML samples sharing this meta-cluster are similar to each other as shown in the red subtree in **(B)**.

From each tube of the AML dataset, using the training samples, we build two templates: one for healthy samples and one for AML. As described in Section 2.6, the HM&M algorithm organizes samples of the same class into a binary template tree whose root represents the class template. The template trees created from the healthy and AML training samples in Tube 6 are shown in Figures 12A,B, respectively. The height of an internal node in the template tree measures the dissimilarity between its left and right children, whereas the horizontal placement of a sample is arbitrary. In these trees, we observe twice as much heterogeneity in the AML samples than among the healthy samples (in the dissimilarity measure), despite the number of healthy samples being five times the number of the AML samples. The larger heterogeneity among AML samples is observed in other tubes as well. The template tree for AML partitions these samples into different subtrees that possibly denote different subtypes of AML. For example, the subtree in Figure 12B that is colored red includes samples (with subject ids 37, 58, 67, 89, and 117) with immunophenotypes of acute promyelocytic leukemia (APL) (discussed later in this section).

Together, the meta-clusters in a healthy template represent a healthy immune profile in the feature space of a tube from which the template is created. We obtained 22 meta-clusters in the healthy template created from Tube 6. The percentage of samples from the training set participating in each of these meta-clusters is shown in Figure 12C. Observe that 6% or more of the healthy samples participate in the nine most common meta-clusters (these constitute the core of the healthy template). The remaining thirteen meta-clusters include populations from a small fraction of samples. These populations could correspond to biological variability in the healthy samples, variations in the FC experimental protocols, and possibly also from the splitting of populations that could be an artifact of the clustering algorithm.

The AML template created from Tube 6 consists of forty meta-clusters (almost twice the number in the more numerous healthy samples). Figure 12D shows that, unlike the healthy samples, the AML samples are heterogeneous with respect to the meta-clusters they participate in: There are 21 meta-clusters that include cell populations from at least 20% of the AML samples. Some of the meta-clusters common to a large number of AML samples represent non-AML-specific cell populations. For example, Figure 12E shows the average marker expressions of the meta-cluster shown in the blue bar in Figure 12D. This meta-cluster has low to medium side scatter and high CD45 expression and therefore represents lymphocytes (Figure 11). Since lymphocytes are not affected by AML, this meta-cluster does not express any AML-related markers, and hence can be described as HLA-DR^−^CD117^−^CD34^−^CD38^−^, as expected. Figure 12F shows the expression profile of another meta-cluster shown in the red bar in Figure 12D. This meta-cluster consists of five cell populations from five AML samples (with subject ids 37, 58, 67, 89, and 117) and exhibits medium side scatter and CD45 expression and therefore, represents myeloid blast cells. Furthermore, this meta-cluster is HLA-DR^−^CD117^+^CD34^−^CD38^+^, and represents a profile known to be that of APL (53). APL is subtype M3 in the FAB classification of AML (51) and is characterized by chromosomal translocation of retinoic acid receptor-alpha (RARα) gene on chromosome 17 with the promyelocytic leukemia gene (PML) on chromosome 15, a translocation denoted as t(15;17). In the feature space of Tube 6, these APL samples are similar to each other while significantly different from the other AML samples. Our template-based classification algorithm groups these samples together in the subtree colored red in the AML template tree shown in Figure 12B.

### 4.5 Identifying Meta-Clusters Symptomatic of AML

In each tube, we register meta-clusters across the AML and healthy templates using the mixed edge cover (MEC) algorithm. Meta-clusters in the AML template that are not matched to any meta-clusters in the healthy template represent the abnormal, AML-specific immunophenotypes while the matched meta-clusters represent healthy or non-AML-relevant cell populations. Table 2 lists several unmatched meta-clusters indicative of AML from different tubes. As expected, every unmatched meta-cluster displays medium side scatter and CD45 expression characteristic of myeloid blast cells, and therefore, we omit FS, SS, and CD45 values in Table 2. We briefly discuss the immunophenotypes represented by each AML-specific meta-cluster in each tube, omitting the isotype control Tube 1 and unstained Tube 8.

**Table 2:** **Some of the meta-clusters characteristic of AML for the 23 AML samples in the training set**.

Tube	Marker expression	#Samples	Fraction of cells
2	Kappa^low^Lambda^low^CD19^+^CD20^−^	5	63% (±6.8)
3	CD7^+^CD4^−^CD8^−^CD2^−^	4	18.0% (±4.8)
4	CD15^−^CD13^+^CD16^−^CD56^−^	17	16.6% (±6.9)
4	CD15^−^CD13^+^CD16^−^CD56^+^	8	11.1% (±5.7)
5	CD14^−^CD11c^−^CD64^−^CD33^+^	10	13.5% (±5.2)
5	CD14^−^CD11c^+^CD64^−^CD33^+^	18	10.8% (±3.8)
5	CD14^low^CD11c^+^CD64^low^CD33^+^	6	13.8% (±4.3)
6	HLA-DR^+^CD117^+^CD34^+^CD38^+^	11	13.3% (±2.6)
6	HLA-DR^+^CD117^±^CD34^+^CD38^+^	13	17.3% (±6.6)
6	HLA-DR^−^CD117^±^CD34^−^CD38^+^	5	12.9% (±4.7)
7	CD5^−^CD19^+^CD3^−^CD10^−^	3	12.3% (±2.4)
7	CD5^+^CD19^−^CD3^−^CD10^−^	3	10.0% (±8.5)
7	CD5^−^CD19^−^CD3^−^CD10^+^	1	9.9%

*In the second column, “−,” “low,” and “+” denote very low, low, and high, abundance of a marker, respectively, and ± denotes a marker that is positively expressed by some samples and negatively expressed by others. The number of samples participating in a meta-cluster is shown in the third column. The average fraction of cells in a sample participating in a meta-cluster and the SD are shown in the fourth column*.

**Figure 13:**
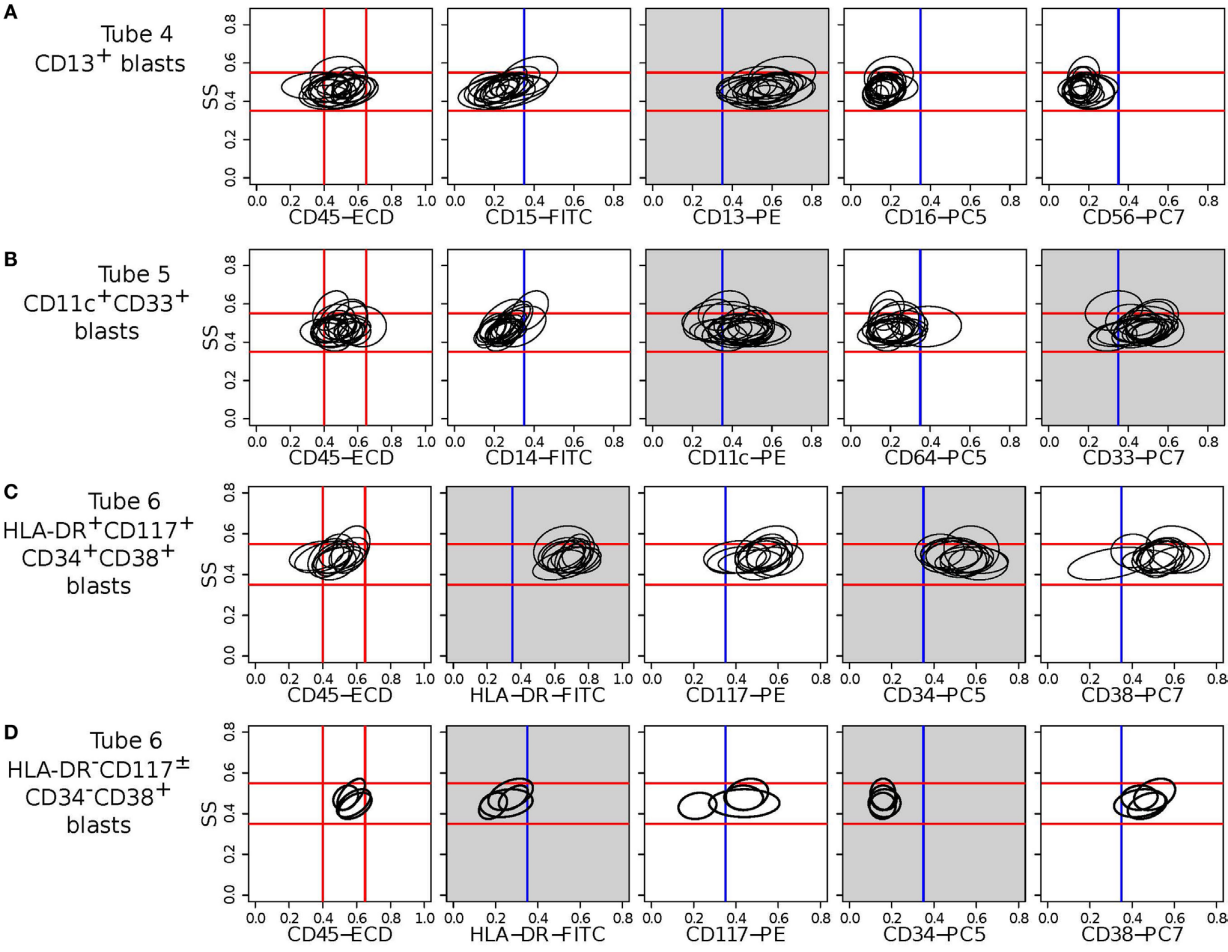
**Bivariate contour plots (side scatter vs. individual marker) for two meta-clusters (one in each row) indicative of AML**. The ellipses in a subplot denote the 95th quantile contour lines of cell populations included in the corresponding meta-cluster. Myeloblast cells have medium side scatter (SS) and CD45 expressions. The red lines indicate approximate myeloblast boundaries (located on the left-most subfigures in each row and extended horizontally to the subfigures on the right) and confirm that these meta-clusters represent immunophenotypes of myeloblast cells. Blue vertical lines denote the ± boundaries of a marker. Gray subplots show contour plots of dominant markers defining the meta-cluster in a row. **(A)** CD13^+^CD56^–^ meta-cluster shared by 17 AML samples in Tube 4. **(B)** CD4^–^CD11c^+^CD64^–^CD33^+^ meta-cluster shared by 18 AML samples in Tube 5. **(C)** HLA-DR^+^CD117^+^CD34^+^CD38^+^ meta-cluster shared by 11 AML samples in Tube 6. **(D)** HLA-DR^–^CD117^±^CD34^–^CD38^+^ meta-cluster shared by 5 AML samples in Tube 6. The last meta-cluster is indicative of acute promyelocytic leukemia (APL).

Tube 6 is the most important panel for diagnosing AML since it includes several markers expressed by AML blasts. HLA-DR is an MHC class II cell surface receptor complex that is expressed on antigen-presenting cells, e.g., B cells, dendritic cells, macrophages, and activated T cells. It is expressed by myeloblasts in most subtypes of AML except M3 and M7 (54). CD117 is a tyrosine kinase receptor (c-KIT) expressed in blasts of some cases (30–100%) of AML (54). CD34 is a cell adhesion molecule expressed on different stem cells and on the blast cells of many cases of AML (40%) (53). CD38 is a glycoprotein found on the surface of blasts of several subtypes of AML but usually not expressed in the M3 subtypes of AML (55). In Tube 6, we have identified two meta-clusters with high expressions of HLA-DR and CD34. One of them also expresses CD117 and CD34, and Figure 13C shows the bivariate contour plots of the cell populations contained in this meta-cluster. The second meta-cluster expresses positive but low levels of CD117 and CD34. These two HLA-DR^+^CD34^+^ meta-clusters together are present in 18 out of the 23 training AML samples. The remaining five samples (subject id: 5, 7, 103, 165, and 174) express HLA-DR^−^CD117^±^CD34^−^CD38^+^ myeloblasts, which is an immunophenotype of APL (53) as was discussed earlier. Figure 13D shows the bivariate contour plots of this APL-specific meta-cluster.

Tube 5 contains several antigens typically expressed by AML blasts, of which CD33 is the most important. CD33 is a transmembrane receptor protein usually expressed on immature myeloid cells of the majority of cases of AML [91% reported in Ref. (56)]. The AML-specific meta-clusters identified from markers in Tube 5 (see Table 2) include CD33^+^ myeloblasts from every sample in the training set. Several of the CD33^+^ populations also express CD11c, a type I transmembrane protein found on monocytes, macrophages, and neutrophils. CD11c is usually expressed by blast cells in acute myelomonocytic leukemia (M4 subclass of AML) and acute monocytic leukemia (M5 subclass of AML) (54). Therefore, the CD14^−^CD11c^+^CD64^−^CD33^+^ meta-cluster could represent patients with M4 and M5 subclasses of AML. We show the bivariate contour plots of this meta-cluster in Figure 13B.

Tube 4 includes several markers usually expressed by AML blasts, of which CD13 is the most important. CD13 is a zinc-metalloproteinase enzyme that binds to the cell membrane and degrades regulatory peptides (57). CD13 is expressed on the blast cells of the majority of cases of AML [95% as reported in Ref. (56)]. Table 2 shows two AML-specific meta-clusters detected from the blast cells in Tube 4. In addition to CD13, eight AML samples express CD56 glycoprotein that is naturally expressed on NK cells, a subset of CD4^+^ T cells and a subset of CD8^+^ T cells. Raspadori et al. (58) reported that CD56 was more often expressed by myeloblasts in FAB subclasses M2 and M5, which covers about 42% of AML cases in a study by Legrand et al. (56). In this dataset, we observe more AML samples expressing CD13^+^CD56^−^ blasts than expressing CD13^+^CD56^+^ blasts, which conforms to the findings of Raspadori et al. (58). Figure 13A shows the bivariate contour plots of the CD13^+^CD56^−^ meta-cluster.

Tube 2 is a B cell panel measuring B cell markers CD19 and CD20, and Kappa (κ) and Lambda (λ), immunoglobulin light chains present on the surface of antibodies produced by B lymphocytes. B cell-specific markers are occasionally co-expressed with myeloid antigens, especially in the FAB M2 subtype of AML [with chromosomal translocation t(8;21)] (54, 59). In Tube 2, we have identified a meta-cluster in the myeloblasts that expresses high levels of CD19 and low levels of Kappa and Lambda. The five samples with subject ids 5, 7, 103, 165, and 174 participating in this meta-cluster possibly belong to the FAB-M2 subtype of AML. Tube 3 is a T cell panel measuring the T cell-specific markers CD4, CD8, CD2, and CD7. Tube 7 is a lymphocyte panel with several markers expressed on T and B lymphocytes and is less important in detecting AML since they are infrequently expressed by AML blasts.

### 4.6 Impact of Each Tube in the Classification

As discussed in the methods section, we build six independent classifiers based on the healthy and AML templates created from Tubes 2–7 of the AML dataset. A sample is classified as an AML sample if the classification score is positive, and as a healthy sample otherwise. Let true positives (TP) be the number of AML samples correctly classified, true negatives (TN) be the number of healthy samples correctly classified, false positives (FP) be the number of healthy samples incorrectly classified as AML, and false negatives (FN) be the number of AML samples incorrectly classified as healthy. Then, we evaluate the performance of each template-based classifier with the well-known four statistical measures: Precision, Recall (Sensitivity), Specificity, and F-value, defined as $\text{Precision}=\frac{\text{TP}}{\text{TP}+\text{FP}}$, $\text{Recall}\;\text{(Sensitivity)}=\frac{\text{TP}}{\text{TP}+\text{FN}}$, $\text{Specificity}=\frac{\text{TN}}{\text{FP}+\text{TN}}$, and $\text{F}-\text{value}=\frac{2(\text{Precision}\times \text{Recall})}{\text{Precision}+\text{Recall}}$. These four measures take values in the interval [0,1], and the higher the values, the better the classifier.

**Figure 14:**
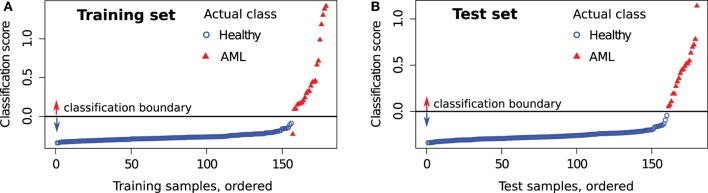
**Average classification score from Tubes 4 to 6 for each sample in the (A) training set and (B) test set**. Samples with scores above the horizontal line are classified as AML and as healthy otherwise. The actual class of each sample is also shown. An AML sample (subject id 116) is always misclassified in the training set, and this is discussed in the text.

First, we evaluate the impact of each tube in the classification of the training samples. For a training sample *X*, the classification score is computed by comparing it with the healthy and AML templates created from the training set after removing *X*. The predicted status of *X* is then compared against true status to evaluate the classification accuracy. Table 3 (left panel) shows various statistical measures for the classifiers defined in Tubes 2–7 of the training set. The classifiers based on Tubes 4–6 have the highest sensitivity because these tubes include several markers relevant to AML diagnosis (53, 54). The number of true negatives TN is high in every tube since the identification of healthy samples does not depend on the detection of AML-specific markers. Hence, specificity is close to one for all tubes. Analogously, FP is low for most tubes, and we observe high precision for most tubes. The F-value is a harmonic mean of precision and recall and denotes the superior classification ability of markers in Tubes 4–6. Averaging scores from all tubes does not improve the sensitivity and F-value dramatically. However, combining Tubes 4–6 gives almost perfect classification with one misclassification for the training set. We plot the average classification scores from Tubes 4 to 6 for the training samples in Figure 14A. The class labels of samples are also shown (blue circles for healthy and red triangles for AML samples).

**Table 3:** **Four statistical measures evaluating the performance of the template-based classification in the training set and test set of the AML data**.

Tubes	Training set				Test set			
	Precision	Recall	Specificity	F-value	Precision	Recall	Specificity	F-value
4	0.94	0.74	0.99	0.83	1.00	0.75	1.00	0.86
5	0.75	0.91	0.96	0.82	0.65	0.85	0.94	0.74
6	1.00	0.70	1.00	0.82	1.00	0.80	1.00	0.89
All (2–7)	1.00	0.74	1.00	0.85	1.00	0.85	1.00	0.92
4–6	1.00	0.96	1.00	0.98	1.00	1.00	1.00	1.00

*The statistical measures are computed for each tube separately and two combinations of tubes*.

**Figure 15:**
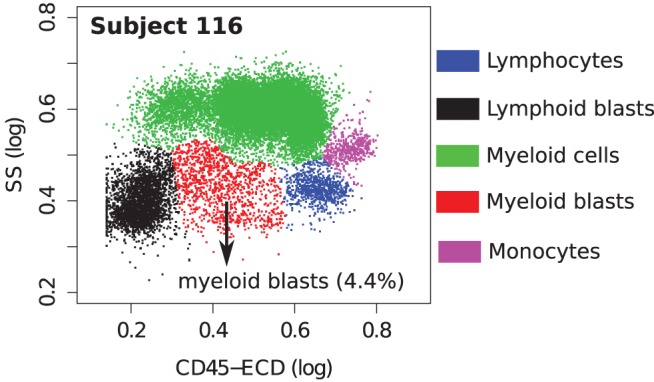
**Cell populations in a samples from subject 116**. This sample contains only 4.4% myeloid blast cells (shown in red).

In Figure 14A, we observe an AML sample (subject id 116) with score below the classification boundary. Figure 15 shows that this sample has only 4.4% myeloid blast cells, which is lower than the minimum 20% AML blasts necessary to recognize a patient to be AML-positive according to the WHO guidelines (60) (the FAB threshold is even higher, at 30%). Hence, this is either a rare case of AML, or one with minimal residual disease after therapy, or perhaps it was incorrectly labeled as AML in the training set. Subject 116 was classified with the healthy samples by methods in other published work (26).

### 4.7 Classifying Test Samples

Now we turn to the test samples. For each tube, we compute the classification score for each sample in the test set using templates created from the training set and applying equation (3). Since the average classification score from Tubes 4 to 6 performs best for the training set, we use it as a classifier for the test set as well. Since the status of test samples was released after the DREAM6/FlowCAP2 challenge, we can determine the classification accuracy of the test samples. Figure 14B shows the classification scores of the test samples, where samples are placed in ascending order of classification scores. In Figure 14B, we observe perfect classification in the test set. Similar to the training set, we tabulate statistical measures for the classifiers in Table 3.

When classifying a sample *X*, we assume the null hypothesis: *X* is healthy (non-leukemic). The sample *X* receives a positive score if it contains AML-specific immunophenotypes, and the higher the score, the stronger the evidence against the null hypothesis. Since Tube 1 (isotype control) does not include any AML-specific markers, it can provide a background distribution for the classification scores. In Tube 1, 174 out of 179 training samples have negative classification scores, but five samples have positive scores, with values less than 0.2. In the best classifier designed from Tubes 4 to 6, we observe that two AML-positive samples in the training set and three AML-positive samples in the test set have scores between 0 and 0.2. The classifier is relatively less confident about these samples; nevertheless, the p-values of these five samples (computed from the distribution in Tube 1) are still small (<0.05), so that they can be classified as AML-positive. The rest of the AML samples in the training and test sets have scores greater than 0.2, and the classifier is quite confident about their status (*p*-value zero).

Four AML samples in the test set (ids 239, 262, 285, and 326) were subclassified as APL by comparing against distinct template trees for APL and the other AML samples in the training set (cf. Figure 12B).

Finally, we state the computational times required on an iMac with four 2.7-GHz cores and 8-GB memory. Our code is in R. Consider a single tube with 359 samples in it. The *k*-means clustering of all samples took 1 h, primarily because we need to run the algorithm multiple times (about ten on the average) to find the optimal value of the number of clusters. Creating the healthy template from 156 samples in the training set required 10 s on one core, and the AML template for 23 AML samples took 0.5 s on one core. Cross validation (leave one out) of the training set took 30 min, and computing the classification score for the 180 test samples took 15 s, both on four cores. We could have reduced the running time by executing the code in parallel on more cores. We have made the dominant step, the *k*-means clustering of all the samples with an optimal number of clusters, faster using a GPU, reducing the total time to a few minutes.

## 5 Conclusion

We have described a set of algorithms for feature selection in a collection of flow cytometry samples by identifying immunophenotypes (cell populations characterizing subsets of the samples that express certain markers and not others). The immunophenotypes are obtained from statistical summaries of similar cell populations in all of the samples. We have used these immunophenotypes to hierarchically organize the samples via a template tree, to identify variations in the samples at multiple levels, and to use them for robust and efficient classification.

We report results from two sets of FC data. We show that a collection of healthy samples from different individuals over a number of days are classified by our approach to identify technical replicates, temporal replicates, and individual replicates. We also show that this identification is not obtained using a different method that uses the earth mover’s distance and UPGMA for constructing a template tree. The second set of data represents phenotypically heterogeneous subtypes of AML samples, where we identified thirteen phenotypes corresponding to the different subtypes. Here we were able to distinguish the APL subtype from other AML subtypes, but other markers not included in the study would be necessary to distinguish other subtypes of AML.

We have assembled our algorithms for the several steps of FC data analysis into a package called flowMatch and made it available as an open-source R package in Bioconductor.^2^ The flowMatch package has been downloaded more than 2,000 times since February 2015. We have also employed various components of this package to analyze other FC datasets and have published the results in peer-reviewed journals and conferences (19, 20, 44).

Stabilizing variance, clustering, matching clusters, and creating templates are general concepts with applications to other areas of biotechnology. Therefore, the algorithms in the flowMatch package can be applied – with simple modifications – to problems outside of flow cytometry. We have already applied the variance stabilization framework to microarray data and compared the results with a state-of-the-art software developed for microarrays (24). Likewise, other algorithms have applications to problems from microarrays, ChIp-Seq, etc. In particular, FC data projected on a lower dimension have considerable similarities with images from traditional photography and bio-imaging technologies such as imaging cytometry, magnetic resonance imaging (MRI), etc., and hence, algorithms in flowMatch could be used to analyze images from these applications.

## Author Contributions

AA, BR, and AP participated in the discussions to conceive the study, designed the algorithms and experiments, and were involved in writing the manuscript. Additionally, AA implemented the algorithms and created the flowMatch and flowVS software. BR created the Purdue healthy subject dataset. All the three authors read and approved the final manuscript.

## Conflict of Interest Statement

The authors declare that the research was conducted in the absence of any commercial or financial relationships that could be construed as a potential conflict of interest.

## Footnotes

^1^http://flowcap.flowsite.org/

^2^http://www.bioconductor.org/
